# A Data-Driven Approach to Responder Subgroup Identification after Paired Continuous Theta Burst Stimulation

**DOI:** 10.3389/fnhum.2017.00382

**Published:** 2017-08-04

**Authors:** Tonio Heidegger, Onno Hansen-Goos, Olga Batlaeva, Onur Annak, Ulf Ziemann, Jörn Lötsch

**Affiliations:** ^1^Clinic of Neurology, Goethe-University Frankfurt am Main, Germany; ^2^Institute of Clinical Pharmacology, Goethe-University Frankfurt am Main, Germany; ^3^Department of Neurology and Stroke, and Hertie Institute for Clinical Brain Research, Eberhard-Karls University Tübingen Tübingen, Germany; ^4^Project Group Translational Medicine and Pharmacology TMP, Fraunhofer Institute for Molecular Biology and Applied Ecology IME Frankfurt am Main, Germany

**Keywords:** transcranial magnetic stimulation, paired continuous theta burst stimulation, subject selection, heterogeneity of plasticity effects, data science

## Abstract

**Background**: Modulation of cortical excitability by transcranial magnetic stimulation (TMS) is used for investigating human brain functions. A common observation is the high variability of long-term depression (LTD)-like changes in human (motor) cortex excitability. This study aimed at analyzing the response subgroup distribution after paired continuous theta burst stimulation (cTBS) as a basis for subject selection.

**Methods**: The effects of paired cTBS using 80% active motor threshold (AMT) in 31 healthy volunteers were assessed at the primary motor cortex (M1) corresponding to the representation of the first dorsal interosseous (FDI) muscle of the left hand, before and up to 50 min after plasticity induction. The changes in motor evoked potentials (MEPs) were analyzed using machine-learning derived methods implemented as Gaussian mixture modeling (GMM) and computed ABC analysis.

**Results**: The probability density distribution of the MEP changes from baseline was tri-modal, showing a clear separation at 80.9%. Subjects displaying at least this degree of LTD-like changes were *n* = 6 responders. By contrast, *n* = 7 subjects displayed a paradox response with increase in MEP. Reassessment using ABC analysis as alternative approach led to the same *n* = 6 subjects as a distinct category.

**Conclusion**: Depressive effects of paired cTBS using 80% AMT endure at least 50 min, however, only in a small subgroup of healthy subjects. Hence, plasticity induction by paired cTBS might not reflect a general mechanism in human motor cortex excitability. A mathematically supported criterion is proposed to select responders for enrolment in assessments of human brain functional networks using virtual brain lesions.

## Introduction

Noninvasive brain stimulation (NIBS) techniques have been widely used for investigating human brain functions and for improving deficits following neuronal damage (Davis and van Koningsbruggen, 2013). Based on early experiments showing the possibility to alter cortical functions (Rasmussen and Penfield, 1947), repetitive transcranial magnetic stimulation (rTMS; Barker et al., 1985) has evolved into a common tool to alter cortical neuronal activity and excitability by inducing neuroplasticity changes (Lefaucheur, 2009). Depending on the stimulation parameters, NIBS can be used either to increase the cortical excitability, referred to as long-term potentiation (LTP)-like changes, or to decrease cortical excitability, known as long-term depression (LTD)-like changes (Hoogendam et al., 2010). In particular in studies examining the functional contribution of distinct brain regions to cortical networks, LTD-like changes are often used as “virtual lesions” (Siebner and Rothwell, 2003).

LTD-like changes in human (motor) cortex excitability are commonly induced using continuous theta burst stimulation (cTBS), which has been shown to provide robust effects at short intervention time and low stimulation intensity (Huang et al., 2005), using an established intensity of 80% of active motor threshold (AMT). However, changes in neuronal plasticity following cTBS application in humans last significantly shorter than those induced in animal models using corresponding LTD-induction procedures (Malenka and Bear, 2004). Therefore, the use of paired or spaced cTBS (from now on referred to as paired cTBS) was suggested to obtain longer and more pronounced LTD-like changes (Goldsworthy et al., 2012). They applied two trains of cTBS separated by an inter-train interval of 10 min. LTD-like changes could be shown for paired cTBS using stimulation intensities of 70% resting motor threshold (RMT) but were not present when paired trains using 80% AMT were delivered. This was assumed to be due to the muscle contraction prior to cTBS application when assessing AMT (Goldsworthy et al., 2012). However, the induction of LTD-like effects by standard cTBS protocols does not seem to be affected by muscle contractions needed for AMT-assessment prior to stimulation. Moreover, evidence suggests that both, the direction and the extent of cTBS-induced changes differ significantly among subjects (Hamada et al., 2013; Hordacre et al., 2015, 2017; Vallence et al., 2015). This variability can result in the absence of plastic changes, up to paradox effects with cTBS-stimulations producing LTP-induction (Chung et al., 2016).

Although the interindividual variability in cTBS effects may potentially confound group effects of virtual lesions in studies of human brain functions, no systematic investigations of the response distribution have been reported so far. Therefore, the present study pursued the hypothesis that only a fraction of subjects responds to paired cTBS using 80% AMT (cTBS_AMT_) showing the expected LTD-like changes and that mathematically based criteria can be identified to separate responders from non-responders or paradox responders, aimed at providing a rational basis for subject selection in studies employing virtual brain lesions to assesses human brain functional networks. A cohort of randomly selected healthy volunteers was subjected to cortical plasticity induction by means of paired cTBS_AMT_, assessing its effects using single-pulse TMS allowing to compare motor evoked potentials (MEPs) before and after induction of neuronal plasticity based on a previous proposal (Hallett, 2007). The present study looks at paired cTBS, which adds to earlier investigations of the response distribution to transcranial stimulation (Hamada et al., 2013; Vallence et al., 2015; Hordacre et al., 2017), and uses *post hoc* machine learning analyses to identify a cutoff for subject selection.

## Materials and Methods

### Subjects and Study Design

The study followed the Declaration of Helsinki and was approved by the Ethics Committee of the Goethe-University Frankfurt am Main, Germany (protocol number 250/11). Informed written consent from each participating subject had been obtained. Effects of paired continuous theta burst stimulation (cTBS_AMT_) on motor cortical excitability were investigated in 31 healthy right-handed volunteers (mean age ± standard deviation, SD: 25.5 ± 4.1 years; 14 men). The subjects’ actual health was ascertained by medical history and physical examination. Inclusion criteria were age between 18 years and 50 years and no relevant current medical history while exclusion criteria were actual diseases, drug intake within a week except for oral contraceptives, and the presence of contraindications for TMS. Alcohol was prohibited for 24 h before the actual experiments.

**Figure 1:**
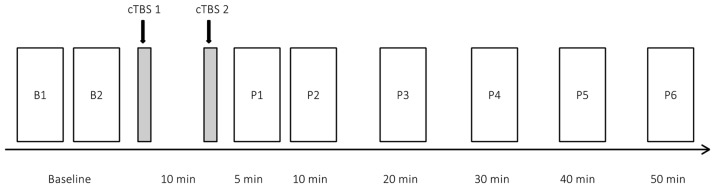
Study design. Rectangles represent blocks of 21 motor evoked potential (MEP) trials measured at baseline (B1, B2) and at as six post interventional blocks after plasticity induction by paired continuous theta burst stimulation (cTBS). Paired cTBS trains (gray bars) were separated by 10 min.

This study employed a single blinded design with respect to the purpose of the stimulation protocol. The experiment (Figure 1) was carried out in the sequence: (i) localization of the individual stimulation site in each subject; (ii) determination of the individual AMT; (iii) baseline recordings of MEP; (iv) induction of neural plasticity; and (v) post-interventional recordings of MEPs. MEPs were recorded in blocks of 21 trials each, delivered at intervals of 10 s (10% random variance) in-between trials. Two blocks were recorded at baseline (B1, B2) and six post-interventional recordings took place at 5 min (P1), 10 min (P2), 20 min (P3), 30 min (P4), 40 min (P5) and 50 min (P6) after completion of the second cTBS_AMT_ train. Measurements were carried out during late morning until early afternoon.

During the experiments, subjects were seated in a comfortable reclining chair with their arms and hands lying relaxed on the armrests. All measurements were conducted during complete voluntary muscle relaxation monitored audio-visually by high-gain (50 μV/Div) electromyography (EMG) and supervision by an additional experimenter. Subjects were told to restrain from movements between the measurements and to remain in a relaxed state of consciousness.

### Transcranial Magnetic Stimulation and Signal Acquisition

#### Localization of the Stimulation Site and Electromyography Recording

The effects of TMS were assessed at the primary motor cortex (M1) corresponding to the representation of the first dorsal interosseous (FDI) muscle of the left hand. The correct localization was identified using MEPs recorded via surface electromyography wafer electrodes (EMG) from the resting FDI muscle of the left hand (active electrode) and the proximal phalanx of the index finger (reference electrode). The raw EMG signal was amplified, filtered (bandpass of 20–2000 Hz; Counterpoint Mk2, Dantec Elektronik, Skovlunde, Denmark), analog-to-digital converted at a sampling rate of 5 kHz (CED Micro 1401; Cambridge Electronic Design, Cambridge, UK) and stored in a computer for online visual inspection and offline analysis.

Focal TMS of the hand area of the right M1 was performed with a figure-of-eight coil (Cool-B65, MagVenture, diameter of each wing 65 mm) connected to a MagPro X100 magnetic stimulator (MagVenture, Farum, Denmark) using a monophasic current waveform to induce posterior-anterior current in the brain. The optimal coil position over the hand area of the right M1 area for eliciting MEPs in the left FDI muscle was defined as the site where TMS at suprathreshold stimulus intensity consistently produced the largest MEP. This “hotspot” was marked with a soft-tipped pen on a swimming cap on the subject’s head to assure constant placement of the coil throughout the measurements. The coil was held tangential to the scalp with the handle pointing backwards and 45° away from the mid-line. This orientation induced a lateral-posterior to medial-anterior current in the brain that activated the corticospinal system preferentially trans-synaptically via horizontal cortico-cortical connections (Di Lazzaro et al., 2008).

AMT was defined to the nearest 1% of maximum stimulator output (MSO) as the lowest stimulus intensity which elicited small MEPs (≥200 μV) in at least five out of ten consecutive trials during a slight tonic contraction (approximately 20% of the maximal strength) of the left FDI muscle, using the relative frequency method (Groppa et al., 2012). The grand average AMT across all subjects was 42.7 ± 7.4% (mean ± SD) of MSO. The stimulation intensity was then adjusted to evoke MEPs in the left FDI muscle with peak-to-peak amplitudes of 1 ± 0.3 mV. This intensity was used for all subsequent MEP recordings in the same subject. The grand average MEP 1 mV across all subjects was 55.1 ± 12.2% of MSO.

#### Induction of Neuronal Plasticity using cTBS

cTBS over the right M1 was delivered by means of a MagPro X100 magnetic stimulator connected to a 65 mm figure-of-eight coil (Cool-B65, MagVenture, Farum, Denmark) using a biphasic current waveform (ap—pa) with the coil placed tangentially to the scalp with the handle pointing backwards and 45° away from the mid-line. Stimulus intensity was set at 80% of AMT; the grand average across all subjects was 34.2 ± 5.9% of MSO. A total of 600 pulses was delivered using an established stimulation pattern with three pulses at 50 Hz and bursts repeated at every 200 ms (Huang et al., 2005). As applied previously (Goldsworthy et al., 2015), two 40s-cTBS trains were delivered at an interval of 10 min.

### Data Analysis

#### Pre-Processing of MEP Data

Single trials that contained real-time audio-visually detected background muscle activation prior to stimulation and the first trial of every recording block were excluded from the analysis. The remaining trials were averaged separately for each subject and time point. Subsequently, the two baseline blocks (B1, B2) were condensed to a single baseline (B) by averaging. The data acquired post-interventionally during the blocks P1 to P6 were normalized to each subject’s individual baseline MEP amplitude. Data were then expressed as the percentage of B. This resulted in six post-interventional data points for each subject, where values >100% indicated a MEP facilitation while values <100% indicated a MEP depression. As MEP depression after paired cTBS has been reported to last at least 60 min (Goldsworthy et al., 2015), data covering the post interventional interval from 5 min to 50 min was averaged for every single subject to obtain a global measure of overall MEP change after plasticity induction.

#### Group Pattern Analysis of MEP Response Data

The global measure of overall MEP change was analyzed for group pattern using the R software package (version 3.3.1 for Linux; R Development Core Team, 2008)^1^ on an Intel Xeon^®^ computer running on Ubuntu Linux 16.04.1. The probability density function (PDF) of the above described MEP changes was analyzed using the Pareto Density Estimation (PDE), which is a kernel density estimator particularly suitable for the discovery of groups in the data (Ultsch, 2003). This revealed a mixture of different distributions that could be modeled with a Gaussian mixture model (GMM) as $p(x)=\sum_{i\;=\;0}^{M}w_{i}N(x|m_{i},s_{i})$ where *N(x|m_i_, s_i_)* denotes Gaussian probability densities (component, mode) with means, *m_i_* and standard deviations, *s_i_* while *w_i_* denotes the mixture weights controlling the relative contribution of each component Gaussian to the overall distribution, which add up to a value of 1 and *M* denotes the number of components in the mixture. GMM fitting was performed with our R package “AdaptGauss”^2^ (Ultsch et al., 2015). This interactive tool allows to visually adjust the fit, i.e., the numerical values could be optimized interactively with the root mean square error between empirical distribution (PDE) and GMM as the fit criterion. To determine the optimum number of components, model optimization was done for *M* = 1–4 components. The final model was selected on the basis of a likelihood ratio test and therefore, the indicator of improvement of the fit was a change in minus two-fold the log likelihood (Δ_−2LL_) and the *χ*^2^ approximation with the number of degrees of freedom equal to the difference in the number of parameters between two models was applied to judge statistical significance, which with an α-level set at 0.05 implies a significance criterion of at least Δ_−2LL_ < 3.84. In addition, the quality of the model to fit the data distribution was assessed by visual inspection of the fit and of a derived quantile-quantile plot (QQ-plot) of the observed and predicted data distributions. Subject association to the identified subgroups of cTBS responses was obtained using the Bayes’ Theorem (McGrayne, 2011) that provided the probability that an individual observation belongs to mode *i* calculated as the posterior probability as the basis to draw the decision boundary between Gaussian modes located at the intersections of the single components’ distributions.

Subsequently, time courses of the MEP changes after plasticity induction by paired cTBS were submitted to analysis of variance for repeated measures (rm-ANOVA), with “block” as within-subject factor (seven levels: averaged baselines, P1, P2, P3, P4, P5 and P6) and “group” as between-subjects factor with the number of levels corresponding to the result of the GMM analysis. Additional between-subjects factors were “sex” (two levels: female and male) and “time of day” (two levels: morning and afternoon). The Greenhouse-Geisser correction of the degrees of freedom (Greenhouse and Geisser, 1959) was applied where as indicated by a positive Mauchly’s test for (non-)sphericity (Mauchly, 1940). To determine the time points at which MEP amplitudes differed significantly from the baseline, *post hoc* comparisons were performed using paired *t*-tests (Student, 1908) against baseline.

As the identification of responders, i.e., subjects displaying a reduction in motor cortical excitability following paired cTBS, was considered of major importance for subject selection for studies employing virtual brain lesions to assess human brain functional networks, responder identification was performed again using a second analytical approach. This exclusively addressed the desired reductions in MEP amplitudes following cTBS, calculated as 100 − MEP [%], while all values >100% were regarded as absent response (0%). To identify responding subjects, these data were submitted to a computed ABC analysis (Ultsch and Lötsch, 2015). This is an item categorization technique originally developed in economical sciences (Pareto, 1909; Juran, 1975) to search for the minimum possible effort that gives the maximum yield. It was used presently as a selective inventory category technique that can be used to identify subjects who promise to display a large response to paired cTBS, probably improving the outcome of experiments in terms of reaching large effect sizes. The ABC technique is a numeral method to classify such responses into separate categories. In principle, it aims at dividing any set of positive data, here the cTBS effects, into three distinct subsets called “A”, “B” and “C”. Subsets “A” and “B” comprise profitable values, i.e., “the important few”, whereas subset “C” comprises non-profitable values, i.e., “the trivial many”. The latter can be regarded as not contributing significantly to the distribution and can therefore be neglected. The calculations were done using our R package “ABCanalysis”^3^ (Ultsch and Lötsch, 2015).

## Results

All 31 subjects finished the study without experiencing any noticeable side effect of TMS. Baseline MEP data did not statistically significantly differ between the two blocks (B1: 0.99 ± 0.14 mV, B2: 0.99 ± 0.26 mV; mean ± SD; *t*-test: *t* = −0.05, *p* = 0.996). The averaged baseline MEP amplitude was 0.99 ± 0.17 mV.

**Figure 2:**
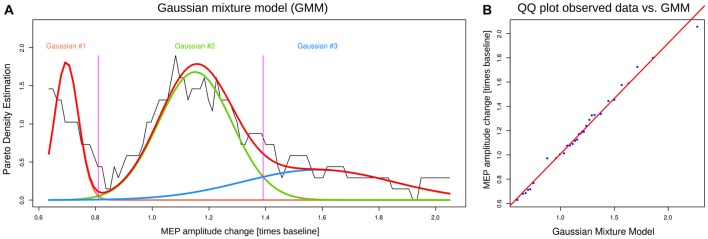
Distribution of the post interventional overall MEP amplitude change, obtained as the individual average of the MEP amplitude changes compared to baseline acquired at six time points (P1…P6) during the post interventional measurements. **(A)** The density distribution is presented as probability density function (PDF), estimated by means of the pareto density estimation (PDE; Ultsch, 2003; black line). A Gaussian mixture model (GMM; Equation 1; GMM given as $p(x)=\sum_{i\;=\;0}^{M}w_{i}N(x|m_{i},s_{i})$), was fit to the data (red line), for which the optimum number of mixes was found to be *M* = 3. Subject distribution among the obtained three Gaussians (green, orange and blue colored lines) was *n* = 6, *n* = 18 and *n* = 7 for Gaussian 1–3, respectively, starting from the left. The Gaussian modes correspond to the subgroups of “responders” (Gaussian #1, left), “non-responders” (Gaussian #2, middle) and “paradox responders” (Gaussian #3, right). **(B)** QQ-plot of the overall MEP amplitude change vs. the GMM. The figure has been created using the R software package (version 3.3.1 for Linux; R Development Core Team, 2008; http://CRAN.R-project.org/). In particular, the GMM analysis was performed and plotted using our R package “AdaptGauss” (Ultsch et al., 2015; http://cran.r-project.org/package=AdaptGauss).

The distribution of MEP change after plasticity induction, obtained as the individual average changes in MEP amplitude from baseline acquired at the six time points (P1…P6) during the post-interventional measurements, could be described with a GMM composed of *M* = 3 Gaussians (Figure 2). This was supported by a significant likelihood ratio test against a GMM with *M* = 2 modes at *p* < 0.01 while adding a fourth Gaussian mode failed to provide a significant improvement of the fit (likelihood ratio test: *p* > 0.05). The tri-modal distribution model (Table 1) parted the cohort into a subgroup of “responders” (*n* = 6), who according to the Bayesian decision border (Figure 2) exhibited an overall MEP-reduction to a value of 80.9% or less (average across all post-cTBS acquired MEP amplitudes), an additional subgroup of “paradox responders” (*n* = 7) separated by a Bayesian decision border at 139.1% from the largest subgroup of “non-responders” (*n* = 18) located around 100% MEP.

**Table 1:** Values of variables obtained following modeling of the distribution of the post interventional overall MEP amplitude change, obtained as the individual average of the motor evoked potential (MEP) amplitude changes compared to baseline acquired at six time points (P1…P6) during the post interventional measurements, by means of the Gaussian mixture model (GMM given as $p(x)=\sum_{i\;=\;0}^{M}w_{i}N(x|m_{i},s_{i})$, for which the optimum number of mixes was found to be *M* = 3 (Figure 2), where *m_i_*, *s_i_* and *w_i_* are the parameters mean, standard deviation and relative weight of each of the Gaussians, *i*, respectively.

GMM parameter	*i* = 1	*i* = 2	*i* = 3
	(first Gaussian)	(2nd Gaussian)	(3rdGaussian)
*m_i_* [% change from baseline]	69.7	115.1	158.4
*s_i_*	4.2	13	26.4
*w_i_*	0.19	0.55	0.26
Bayesian decision limit [%]	80.9		139.1	

**Figure 3:**
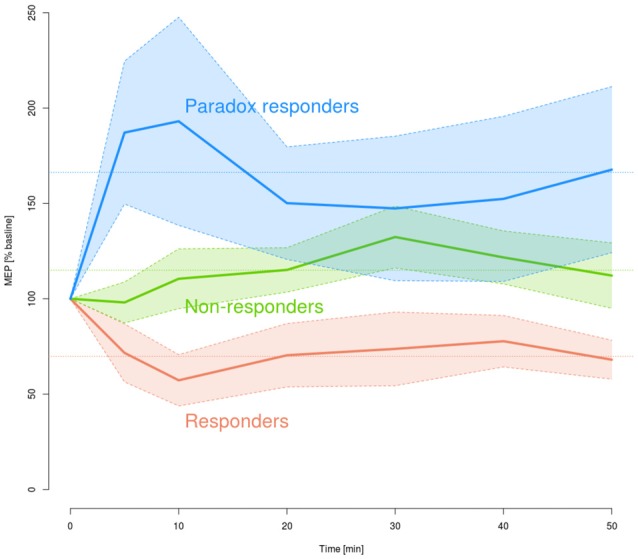
Time courses of the MEP amplitude changes after plasticity induction by paired cTBS, means (solid lines) and 95% confidence intervals (shaded regions), separately for the different responder subgroups (red: responder, *n* = 6, green: non-responder, *n* = 18, blue: paradox responder, *n* = 7). The horizontal lines indicate the averaged MEP amplitudes over all time points of post-cTBS measurements for the three subgroups.

The identified subgroups differed with respect to MEP amplitude changes during all post-interventional time points after plasticity induction using paired cTBS_AMT_ (Figure 3). This was indicated by a significant main effect of the factor “group” in the rm-ANOVA (*df* = 2.28, *F* = 78.7, *p* = 3.2112 · 10^−12^) and a significant statistical interaction “block” by “group” (*df* = 6.87, 96.184, *F* = 3.608, *p* = 0.001827; effect “block” not significant). The factors “sex” and “time of day” did not show a significant effect. In addition, a separate rm-ANOVA only in the “responders” subgroup “block” (seven levels) as within-subject factor confirmed a significant depression of post-interventional MEP amplitudes (*df* = 6.30, *F* = 3.086, *p* = 0.018). *Post hoc* analyses showed a significant MEP-decrease compared to baseline for all post-interventional time points (*t*-tests: *p* ≤ 0.044 for all). However, the latter indicates that differences do not pass α correction and indeed, the distinction between “non-responders” and “paradox-responders” is weak as also indicated by visual inspection of the PDF (Figure 2).

As for subject selection for studies employing virtual brain lesions to assess human brain functional networks, the “responders” group is of greatest interest. Therefore, “responder” subgroup separation from the rest of candidate subjects was reassessed by submitting the cTBS effects, defined as a post-interventional decrease in MEP-amplitude, to ABC analysis. This indicated *n* = 6 subjects located in ABC sets “A” and “B”, i.e., in the sets where the contribution to the effect of interest is important or at least appropriate to the effects of subject inclusion whereas again, *n* = 25 subjects were placed in ABC set “C” where only trivial contributions to the effect were seen. Hence, the ABC analysis was in agreement with the GMM analysis in identifying *n* = 6 clear responders among the *n* = 31 candidate subjects, which agreed with the GMM result.

## Discussion

The present results provide further evidence for a large interindividual variability of the responses to paired cTBS_AMT_ (Goldsworthy et al., 2012). Strong and long-lasting depressive effects of paired cTBS_AMT_ could be induced; however, only in a subgroup of the study population. The analysis of the probability density distribution of the cTBS effects provided a clear separation of the responder subgroup (Figure 2), which was verified by the results of an item categorization analysis. The observed group patterns could not be attributed to distinguishing features of the study population or stimulation procedure, i.e., the subjects’ sex or the time of stimulation. Therefore, present results provide a mathematically supported criterion of responder selection as a robust basis for subject inclusion into studies employing virtual brain lesions to assess human brain functional networks.

The increase in reliability of TMS assessments is an active research topic that has led to a variety of further proposals. A promising path seem to be closed-loop stimulation procedures using EEG as readout to avoid possible confounders at spinal and neuromuscular transmission levels (Zrenner et al., 2016). Further proposals include the development of novel stimulation protocols such as quadripulse theta burst stimulation shown to effectively alter cortical excitability (Jung et al., 2016). If modal distribution was not analyzed, the present data would have appeared as if displaying a high variability of the responses to TMS as reported for paired cTBS_AMT_ (Goldsworthy et al., 2012) and extending to intermittent TBS (iTBS; Schilberg et al., 2017). The high degree of variability extends to clinical settings; for example, studies in the stroke population assessing TMS as an adjunct to rehabilitation are characterized by large variability as well (Butler et al., 2005; Otal et al., 2015). Moreover, the problem of high individual response variability is not limited to TMS. It also concerns other NIBS techniques such as TCDS (Nuzum et al., 2016). Systematic reviews therefore concluded that the reliability of TMS as a tool for changing and measuring cortical excitability may be low and seems to be vulnerable to methodological and other confounders (Beaulieu et al., 2017). Significant group effects occasionally cannot be replicated on the individual level (López-Alonso et al., 2014). Further studies have investigated the response distribution to transcranial stimulation including the separation of responders from non-responders for both cTBS and iTBS (Hamada et al., 2013). In that study in 56 healthy volunteers, subjects showing long MEP-latency differences to different current orientations were significantly more frequent in the responder group (see Figure 5 of Hamada et al., 2013). Furthermore, MEP variability and monophasic MEP latency have been shown in 34 healthy subjects to predict 31% of the individual variability of the response to cTBS (Hordacre et al., 2017). Finally, in 18 healthy volunteers cTBS induced MEP suppression was shown to be greatest at high stimulation intensities (with test stimuli at >150% of RMT; Vallence et al., 2015); stimulus intensity was not addressed in the present study.

By pointing at a distinct subgroup of responders, or of paradox responders, the present results may provide a basis to enroll a selected and therefore more homogenous study population as a promising approach to reduce the problems associated with apparent or true high variability of the responses to NIBS. This primarily applies to the “responder” subgroup that was clearly separated from the rest of the cohort (Figure 2). However, when using the presented criteria (Table 1) for subject selection it needs to be assessed: (i) whether the selection is stable over time, i.e., the intra-individual variability of the responses; and (ii) the applicability of the present motor criterion, i.e., MEP, to responses in the sensory system (e.g., somatosensory effects on S2) has to be investigated. Subgroup separation applies to a lower degree to the separation of “paradox responders”. A separate subgroup is suggested by the trimodal GMM; however, the separation is weaker than that of the “responder” subgroup (Figure 2) and would not pass a confirmatory analysis of variance with a correction. Hence, while “paradox” responders have some support in the present data and the separation reproduces a similar proposal from earlier reports (Chung et al., 2016), the main result of the present analysis is the separation of responders and is therefore consistently addressed solely in the ABC analysis.

While the present results strongly indicate responder subgroups to paired cTBS_AMT_, a limitation of the present assessments relates to the possibility that we cannot unequivocally exclude a partial influence of individual confounders. Specifically, despite the instructions to remain in a relaxed state of consciousness, some subjects might nevertheless have influenced the MEP unintentionally; a possibility emerging from the demonstration that motor cortex excitability can be modulated by mental simulation of movements (Hyde et al., 2017). This is in agreement with studies supporting the hypothesis of MEP being modulated by the prestimulus brain state (Iscan et al., 2016). These general problems were addressed by repeated instructions throughout the experiments combined with medical observation of the study participants.

Using a data-driven approach, present results point at a clear group separation of the response to paired cTBS_AMT_, without presenting a possible neurophysiological reason underlying this distinction among healthy subjects. The results were obtained by applying techniques of machine-learned data analysis and supported by a statistical verification. The observation possibly implies that neuronal plasticity induction by paired cTBS might not reflect a general mechanism in human motor cortex excitability but depend on further yet unknown individual factors. Thus, the development of a mathematically supported criterion, based on the present exploratory assessments, is proposed as a basis for enrolment of highly-selected responders into studies employing virtual brain lesions to assess human brain functional networks. However, this still requires the establishment of the intra-individual stability of responder assignment and the assessment of the applicability of the recent results to cTBS effects on sensory brain areas.

## Author Contributions

JL, UZ, OH-G and TH: conceived and designed the experiments. OH-G, TH, OA and OB: performed the experiments. JL and TH: analyzed the data. JL, TH and OA: wrote the article.

## Conflict of Interest Statement

The authors declare that the research was conducted in the absence of any commercial or financial relationships that could be construed as a potential conflict of interest.

## Footnotes

^1^http://CRAN.R-project.org/

^2^https://cran.r-project.org/package=AdaptGauss

^3^http://cran.r-project.org/package=ABCanalysis
